# Altered peripheral amino acid profile indicate a systemic impact of active celiac disease and a possible role of amino acids in disease pathogenesis

**DOI:** 10.1371/journal.pone.0193764

**Published:** 2018-03-14

**Authors:** Åsa Torinsson Naluai, Ladan Saadat Vafa, Audur H. Gudjonsdottir, Henrik Arnell, Lars Browaldh, Staffan Nilsson, Daniel Agardh

**Affiliations:** 1 Institute of Biomedicine, Department of Microbiology & Immunology, Sahlgrenska Academy, University of Gothenburg, Gothenburg, Sweden; 2 Institute of Clinical Sciences, Sahlgrenska Academy at the University of Gothenburg, Gothenburg, Sweden; 3 Department of Pediatric Gastroenterology, Hepatology and Nutrition, Karolinska University Hospital and Division of Pediatrics, CLINTEC, Karolinska Institute, Stockholm, Sweden; 4 Department of Clinical Science and Education, Karolinska Institute, Sodersjukhuset, Stockholm, Sweden; 5 Department of Mathematical Sciences, Chalmers University of Technology, Gothenburg, Sweden; 6 Diabetes & Celiac Disease Unit, Department of Clinical Sciences, Lund University, Malmö, Sweden; Nathan S Kline Institute, UNITED STATES

## Abstract

**Background:**

We have previously performed a Genome Wide Association and linkage study that indicated a new disease triggering mechanism involving amino acid metabolism and nutrient sensing signaling pathways.

**Objective:**

The aim of this study was to investigate if plasma amino acid levels differed among children with celiac disease compared with disease controls.

**Materials and methods:**

Fasting plasma samples from 141 children with celiac disease and 129 non-celiac disease controls, were analyzed for amino acid levels by liquid chromatography-tandem mass spectrometry (LC/MS). A general linear model using age and experimental effects as covariates was used to compare amino acid levels between children with a diagnosis of celiac disease and controls.

**Results:**

Seven out of twenty-three analyzed amino acids were elevated in children with celiac disease compared with controls (tryptophan, taurine, glutamic acid, proline, ornithine, alanine and methionine). The significance of the individual amino acids do not survive multiple correction, however, multivariate analyses of the amino acid profile showed significantly altered amino acid levels in children with celiac disease overall and after correction for age, sex and experimental effects (p = 8.4 × 10^−8^).

**Conclusion:**

These findings support the idea that amino acids could influence systemic inflammation and play a possible role in disease pathogenesis.

## Introduction

Celiac disease is manifested as an intolerance to gluten in genetically at risk individuals, leading to an autoimmune response to tissue transglutaminase (tTG) [1]. By excluding gluten from the diet the damaged intestinal mucosa is restored in most patients and tTG autoantibody (tTGA) levels normalized [2]. Although gluten and certain HLA-genotypes are necessary for celiac disease to develop, the mechanisms by which autoimmunity can be switched on and off by an external food antigen still remains unresolved.

We previously performed a Genome Wide Associations Study (GWAS) in patients with celiac disease, which pointed towards genes involved in nutrient and amino acid signaling [3]. The differential RNA expression of some of these genes (*PRR5L*, *GLS*, *APPL2* and *INSR*) in celiac disease as compared to disease controls, further indicated possibly disturbed mechanisms in nutrient sensing pathways [3]. Whether this is an effect of an underlying disturbed metabolism caused by a damaged intestinal mucosa or vice versa, still remains to be elucidated. In patients affected by other chronic diseases such as type 2 diabetes [4–7], Alzheimer [8], autism [9] and in psoriasis [10], the amino acid profile have been shown to differ from healthy individuals. Amino acid concentrations also differ by age; adolescents with type 2 diabetes tend to have lower levels as compared with healthy individuals, [11] while levels are increased in adults with type 2 diabetes [12], indicating that studies need to be performed on pediatric and adult patients separately.

Amino acids can be used as energy as well as influence cell metabolism and immunity. A well-studied signaling pathway connecting amino acid signaling and immunity is the Target of Rapamycin (TOR) pathway [13]. Interestingly, several of the genes identified by our GWAS belong to this signaling pathway. We also found a possible connection between extra cellular matrix and celiac disease. Extracellular matrix is a storage reservoir for certain amino acids such as proline and hydroxyproline and these amino acids are used as energy during starving conditions. However, when not starving, the release of these amino acids could be a so called endogenous danger signal [14] for the immune system. We therefore hypothesized that certain amino acids, liberated upon gluten metabolism, are involved in signaling to the immune system, ultimately leading to chronic inflammation [3]. In this study, we aimed to investigate if levels of these amino acids are different in plasma from children with celiac disease compared with controls.

## Materials and methods

### Subjects

Peripheral blood samples were consecutively collected from 141 children with untreated celiac disease and 129 disease controls (Table 1). The aim was to include all children who underwent upper endoscopy at the pediatric gastroenterology units in Malmö, Gothenburg and Stockholm, Sweden between 2010 and 2012[15]. Children were fasting at least 6 hours prior to endoscopy procedure performed in general sedation with propofol. Children being positive for tTGA and showing characteristic villous atrophy of the distal part of duodenum (Marsh ≥2) were defined as having celiac disease whereas tTGA positive children having a Marsh <2 were defined as having potential celiac disease. Children being tTGA negative with a normal biopsy excluding celiac disease were included as disease controls.

**Table 1:** Clinical characteristics according to diagnostic status: Celiac disease and disease controls. Age is presented as mean (SD, min-max). The p-values were calculated using ANOVA and Chi-square test.

	Celiac disease n = 141	Disease control n = 129	P-value
**Age, years**	6.6 (3.8, 1,6–17,8)	12 (4.4, 1.4–18)	6.10 x 10^−12^
**Females, n (%) Male, n (%)**	98 (69.5%)43 (30.5%)	74 (57.4%)55 (42.6%)	0.043

All participants were informed about the study and a parental written consent was obtained for each child. The regional ethical review board (EPN) in Gothenburg Sweden approved the study.

### Sample preparation

Eight different batches were prepared separately. 900 μL of extraction buffer (90/10 v/v methanol:water) including internal standards were added to 100 μL of sample material. The sample was shaken at 30 Hz for 2 minutes in a mixer mill and proteins were precipitated at +4°C on ice. The sample was centrifuged at +4°C, 14 000 rpm, for 10 minutes. 100 μL of supernatant were transferred to a micro vial and solvents were evaporated. Samples were derivatized with AccQ-Tag™ (Waters, Milford, MA, USA). The derivatization was performed according to the manufacturer’s protocol; the dried extracts were re-suspended in 20 μL of 20 mM HCl, and 60 μL of AccQ•Tag Ultra borate buffer were added to each sample for pH adjustment. Finally 20 μL of freshly prepared AccQ•Tag derivatization solution were added and the sample was immediately vortexed for 10 seconds. After mixing, the samples were allowed to stand for 30 minutes at room temperature followed by 10 minutes at 55°C. A 14-point calibration set covering the range from 5 fmol to 5 pmol on column was prepared and analysed in triplicates. Norvaline was used as internal standard in a concentration of 0.5 pmol/μL in both samples and in the calibration set.

### Analysis

Liquid chromatography-tandem mass spectrometry (LC/MS) measurements were performed on a 1290 Infinity system from Agilent Technologies (Waldbronn, Germany), with an Agilent 6460 Triple quadrupole mass spectrometer for MRM-detection. The analysis was performed as follows.

1 μL aliquots of the derivatized samples sample were injected onto a 2.1 x 100 mm, 1.7 μm UHPLC Kinetex C18-column (Phenomenex Torrance, CA, USA) held at 50°C in a column oven. The gradient elution buffers were A (H2O, 0.1% formic acid) and B (acetonitrile, 0.1% formic acid), and the flow-rate was 500 μl min-1; mass spectrometry grade formic acid was purchased from Sigma-Aldrich (St Louis, MO, USA) and HPLC grade acetonitrile from Fisher Scientific (Fair Lawn, NJ, USA). The initial condition (0.1% B) was held up to 0.54 minutes. From 0.54 to 5.5 minutes the proportion of solvent B was linearly increased from 0.1% to 9.1%. From 5.5 minutes, B was increased linearly to reach 21.2% at 7.7 minutes. From 7.7 min to 8.5 min the percentage of B was further increased to 59.6% and held there for 0.5 minutes. To elute the more non-polar compounds, the proportion of solvent B was then rapidly increased to 80% at 9.5 min and kept there for 0.5 minutes. From 10 to 10.5 minutes the column was returned to its initial conditions (0.1% B), and the column was equilibrated for 4.5 minutes before injection of the subsequent sample.

The separated amino acids were detected with an Agilent 6460 triple quadrupole (QqQ) mass spectrometer equipped with a jet stream electrospray source operating in positive ion mode. The jet-stream gas temperature was 325°C with a gas flow rate of 10 L min-1, sheath gas temperature of 325°C, and sheath gas flow of 12 L min-1. The nebulizer pressure was set to 20 psi and the capillary voltage was set at 4 kV. The QqQ was run in Dynamic MRM Mode with 0.5 minute retention time windows and 500 ms cycle scans. MRM transitions for the derivatized amino acids were optimized using MassHunter MS Optimizer software (Agilent Technologies Inc., Santa Clara, CA, USA). The optimized fragmentation voltages varied from 81–119 V and the collision energies from 14–35 V; nitrogen was used as collision gas.

Quantification of the compounds was performed with MassHunter™ Quantitative Analysis QQQ (Agilent Technologies Inc., Santa Clara, CA, USA). Calibrators were used to construct a standard curve by plotting the ratio of analyte area/internal standard area against the corresponding concentrations of the calibrators. The slope and intercept of the standard curve were used to calculate the concentration of the analyte in the samples using the equation y = ax + b, where y is area ratio, a is slope, x is concentration and b is intercept.

### Statistical analysis

A general linear model, using age and experimental effects such as run batch, as covariates were used to compare amino acid levels between children with a diagnosis of celiac disease with control children. Partial correlation coefficients controlling for age and experimental effects were calculated for all pairs of amino acids. We used Hotelling’s Trace test, corrected for age and experimental effects (such as batch effects and sample handling temperature), to analyze the overall difference in amino acid levels.

## Results

In total, seven out of the 23 amino acids tested showed individually nominally significantly higher levels in children with celiac disease compared with controls after correction for age and experimental effects. The most significant was tryptophane (p = 0.004) (Table 2). Furthermore, taurine, glutamic acid, proline, ornithine, alanine and Methionine were also nominally significant for celiac disease (p<0.05). (Table 2). When using permutation analyses for correction of multiple testing, none of the single amino acids remained significant. However, overall levels of amino acids in plasma were significantly associated with celiac disease (p = 8.4 × 10^−8^). No significance was detected for the so called Fischer’s ratio (branched-chain amino acids (leucine, valine, isoleucine) to aromatic amino acids (phenylalanine, tyrosine).

**Table 2:** Multivariate analysis of the difference between children with celiac disease (cases) and controls. Amino acid levels are analyzed using a general linear model with age, sex and experimental effects as covariates. Nominal p-values are presented for diagnosis of celiac disease vs controls, for age of sampling and for sex. Mean levels are reported as concentrations in plasma (pmol/μl).

Amino acid	CD vs. control	Age	Controls	CD
	p-value	p-value	Mean pmol/μl	Mean pmol/μl
Tryptophane	**0.004**	0.01	43.8	45.9
Taurine	**0.005**	1.13E-03	86.9	99.6
Glutamic Acid	**0.015**	0.45	106.3	123.7
Proline	**0.017**	2.63E-10	160.9	156.2
Ornithine	**0.017**	1.28E-05	80.8	83.3
Alanine	**0.045**	7.60E-07	299.9	300.3
Methionine	**0.048**	4.87E-09	16.4	17.0
Phenylalanine	0.075	1.62E-11	48.3	47.7
Glutamine	0.136	0.10	515.4	486.8
Aspartic Acid	0.142	0.36	9.1	12.4
Tyrosine	0.147	8.21E-04	64.0	63.8
Leucine	0.156	5.90E-13	132.7	125.0
Histidine	0.158	5.82E-04	106.7	110.1
Cysteine	0.171	1.76E-07	13.7	14.7
Serine	0.275	0.91	139.9	146.4
Valine	0.485	3.97E-06	194.5	189.6
Lysine	0.562	1.82E-12	125.7	122.7
Glycine	0.57	0.62	216.2	223.6
Threonine	0.709	9.39E-07	93.9	93.4
Citrulline	0.799	7.21E-10	149.6	129.3
Arginine	0.847	3.00E-05	36.8	33.1
Asparagine	0.864	5.46E-04	54.1	54.8
Isoleucine	0.928	9.01E-11	60.3	54.5

CD = Celiac disease. Nominally significant p-values in the CD vs Control group are presented in bold.

Higher levels of amino acids were also strongly associated with age, with higher levels among older children in 18 of the 23 analyzed amino acids. Leucine (p = 5.9 × 10^−13^), lysine (p = 1.82 × 10^−12^), phenylalanine (p = 1.62 × 10^−11^), isoleucine (p = 9.01 × 10^−11^), proline (p = 2.63 × 10^−10^) and citrulline (p = 7.21 × 10^−10^) being the six most significantly affected by age (Table 2).

To investigate the interrelationships between different amino acids we calculated the correlation between each pair of amino acids. Partial correlation coefficients were significant for almost all amino acid pairs (S2 Table). The most significant correlation was between citrulline and proline (correlation coefficient 0.91, p-value < 0.0001).

## Discussion

This study found that children with untreated celiac disease had increased levels of seven amino acids (tryptophan, taurine, glutamic acid, ornithine, proline, alanine and methionine) compared with disease controls. Another important finding was that almost all amino acids correlated with each other and that the overall level of amino acids was different between cases and controls (p = 8.4 × 10^−8^). This could make it difficult to single out any one amino acid which would be important for disease pathogenesis. However, even though the single amino acids are only nominally significant, the fact that proline and glutamic acid was nominally significantly higher in children with celiac disease supports our previous hypothesis that these amino acids, which also are abundant in wheat gluten, could be causal in triggering the disease[3].

**Fig 1:**
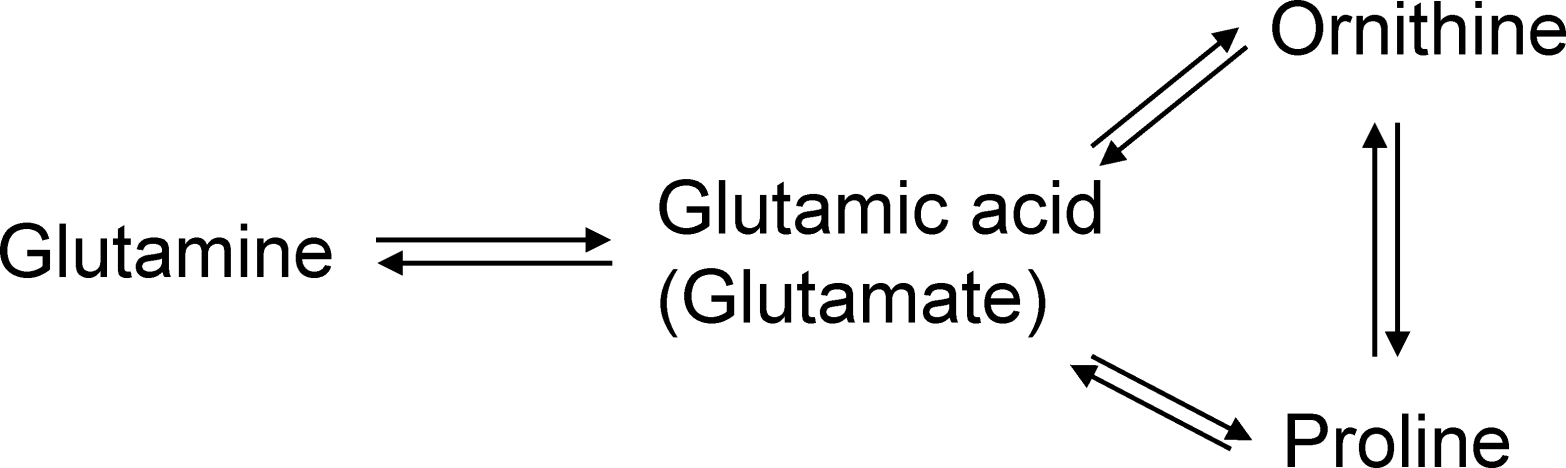
The interrelationship between associated amino acids, proline, ornithine and glutamic acid.

Interestingly, high proline and glutamic acid levels have also been shown to associate with psoriasis [10], another autoimmune disease affecting the skin and high glutamic acid values are associated with the subsequent appearance of the type 1 diabetes associated autoantibodies in young children[16]. Out of the seven amino acids that showed changes in levels, glutamic acid, ornithine and proline are tightly interconnected with each other (Fig 1) Glutamic acid and proline together account for one half or more of the peptide-bound amino acids in wheat gluten[17]. It is possible that children with celiac disease, either eat a larger amount of gluten containing foods, or that they have a slightly impaired metabolism regarding these amino acids and therefore have an elevated level.

Almost all amino acid levels correlated significantly with age, which is in line with a previous study[18]. Younger children had lower levels and older children had higher levels of amino acids. In this study, children with celiac had higher levels at all ages compared with controls of the same age. Lower levels of amino acids in younger children could perhaps indicate higher sensitivity to a disturbance of the amino acid balance. In contrast to children, amino acid levels in a study of adult patients demonstrated lower levels of multiple amino acids in untreated celiac disease compared with healthy adults[19]. This could possibly reflect differences between pediatric and adult celiac disease that could be related to longer duration of inflammation in adult patients resulting perhaps in a relative deficiency in amino acids.

The strength of this study was the large number of patients recruited and the relatively uniform handling of the samples from patients consecutively collected at a limited number of collection sites. The weaknesses of this study were that the controls were older than the cases and that controls were also children with potential gastrological diseases. The cases and controls were significantly different regarding age. The reason for this was that all children who made an upper endoscopy were included in the study and children with celiac disease usually have a lower age at diagnosis.

However, when adjusting for age as well as experimental effects and sample handling procedures, the findings still remained different between children with active celiac disease compared with disease controls.

In conclusion, children with active celiac disease have increased levels of certain amino acids in peripheral blood. Overall these findings supports the possibility that metabolism of amino acids could influence systemic inflammation. However, it is still unclear whether these findings are consequences of the inflammation found in patients with celiac disease or perhaps the results of a genetic predisposition in combination with environmental risk factors. Future studies on samples collected prior to diagnosis are therefore warranted in order to help disentangle the role of amino acid levels in celiac disease pathogenesis.

## Supporting information

S1 Table DATA aminoacids in CD. (XLSX)

S2 Table Partial correlation coefficients. (DOCX)

## Abbreviations

tTG: tissue transglutaminase
tTGA: tissue transglutaminase autoantibody
HLA: human leukocyte antigen
GWAS: Genome Wide Associations Study
*PRR5L* or *Protor-2*: Proline Rich 5 Like
*GLS*: Glutaminase
*APPL2*: Adaptor Protein, Phosphotyrosine Interacting With PH Domain And Leucine Zipper 2
*INSR*: Insulin receptor
