# Supplementary material for: Altered peripheral amino acid profile indicate a systemic impact of active celiac disease and a possible role of amino acids in disease pathogenesis
Source: PLoS One. 2018 Mar 14;13(3):e0193764. doi: 10.1371/journal.pone.0193764 (PMC5851604; doi:10.1371/journal.pone.0193764)
Supplement: S2 Table — (DOCX) [file pone.0193764.s002.docx]

**S2 Table. Partial correlation coefficients.** Calculated between all pairs of amino acids using age and experimental effects as covariates. Significant correlations are marked with ** <0.01 and * <0.05. The strongest correlation (between proline and citrulline) is marked with borders.

|  | Alanine | Arginine | Asparagine | Aspartic Acid | Citrulline | Cystine | Glutamic Acid | Glutamine | Glycine | Histidine | Isoleucine | Leucine | Lysine | Methionine | Ornithine | Phenylalanine | Proline | Serine | Taurine | Threonine | Tryptophane | Tyrosine | Valine |
| --- | --- | --- | --- | --- | --- | --- | --- | --- | --- | --- | --- | --- | --- | --- | --- | --- | --- | --- | --- | --- | --- | --- | --- |
| Alanine | 1,000 |  |  |  |  |  |  |  |  |  |  |  |  |  |  |  |  |  |  |  |  |  |  |
| Arginine | ,168^**^ | 1,000 |  |  |  |  |  |  |  |  |  |  |  |  |  |  |  |  |  |  |  |  |  |
| Asparagine | ,619^**^ | 0,082 | 1,000 |  |  |  |  |  |  |  |  |  |  |  |  |  |  |  |  |  |  |  |  |
| Aspartic Acid | ,257^**^ | 0,057 | ,287^**^ | 1,000 |  |  |  |  |  |  |  |  |  |  |  |  |  |  |  |  |  |  |  |
| Citrulline | ,442^**^ | ,210^**^ | ,280^**^ | 0,093 | 1,000 |  |  |  |  |  |  |  |  |  |  |  |  |  |  |  |  |  |  |
| Cystine | ,158^**^ | ,285^**^ | 0,106 | -0,038 | ,123^*^ | 1,000 |  |  |  |  |  |  |  |  |  |  |  |  |  |  |  |  |  |
| Glutamic Acid | ,222^**^ | -0,017 | ,160^**^ | ,716^**^ | 0,034 | -0,077 | 1,000 |  |  |  |  |  |  |  |  |  |  |  |  |  |  |  |  |
| Glutamine | ,326^**^ | ,256^**^ | ,441^**^ | -,190^**^ | ,266^**^ | ,230^**^ | -,404^**^ | 1,000 |  |  |  |  |  |  |  |  |  |  |  |  |  |  |  |
| Glycine | ,544^**^ | ,139^*^ | ,503^**^ | ,217^**^ | ,252^**^ | ,145^*^ | ,217^**^ | ,171^**^ | 1,000 |  |  |  |  |  |  |  |  |  |  |  |  |  |  |
| Histidine | ,493^**^ | 0,080 | ,685^**^ | ,342^**^ | ,227^**^ | 0,057 | ,255^**^ | ,341^**^ | ,260^**^ | 1,000 |  |  |  |  |  |  |  |  |  |  |  |  |  |
| Isoleucine | ,201^**^ | ,144^*^ | ,243^**^ | ,354^**^ | ,177^**^ | 0,005 | ,291^**^ | -0,030 | 0,029 | ,354^**^ | 1,000 |  |  |  |  |  |  |  |  |  |  |  |  |
| Leucine | ,176^**^ | 0,050 | ,314^**^ | ,393^**^ | ,142^*^ | -0,033 | ,319^**^ | -0,065 | 0,047 | ,470^**^ | ,892^**^ | 1,000 |  |  |  |  |  |  |  |  |  |  |  |
| Lysine | ,473^**^ | ,223^**^ | ,548^**^ | ,317^**^ | ,357^**^ | ,216^**^ | ,195^**^ | ,233^**^ | ,336^**^ | ,576^**^ | ,375^**^ | ,486^**^ | 1,000 |  |  |  |  |  |  |  |  |  |  |
| Methionine | ,612^**^ | ,339^**^ | ,621^**^ | 0,090 | ,364^**^ | ,218^**^ | -0,043 | ,455^**^ | ,425^**^ | ,517^**^ | ,330^**^ | ,326^**^ | ,562^**^ | 1,000 |  |  |  |  |  |  |  |  |  |
| Ornithine | ,537^**^ | -,215^**^ | ,610^**^ | ,354^**^ | ,313^**^ | -0,033 | ,286^**^ | ,203^**^ | ,506^**^ | ,478^**^ | ,235^**^ | ,357^**^ | ,558^**^ | ,400^**^ | 1,000 |  |  |  |  |  |  |  |  |
| Phenylalanine | ,347^**^ | 0,103 | ,463^**^ | ,446^**^ | ,152^*^ | 0,025 | ,280^**^ | 0,056 | ,213^**^ | ,566^**^ | ,613^**^ | ,727^**^ | ,514^**^ | ,511^**^ | ,409^**^ | 1,000 |  |  |  |  |  |  |  |
| Proline | ,565^**^ | ,215^**^ | ,383^**^ | ,120^*^ | ,910^**^ | ,170^**^ | 0,071 | ,324^**^ | ,283^**^ | ,352^**^ | ,188^**^ | ,169^**^ | ,398^**^ | ,472^**^ | ,397^**^ | ,248^**^ | 1,000 |  |  |  |  |  |  |
| Serine | ,489^**^ | ,120^*^ | ,665^**^ | ,370^**^ | ,268^**^ | 0,057 | ,318^**^ | ,268^**^ | ,628^**^ | ,499^**^ | ,210^**^ | ,222^**^ | ,445^**^ | ,425^**^ | ,508^**^ | ,287^**^ | ,318^**^ | 1,000 |  |  |  |  |  |
| Taurine | ,281^**^ | 0,115 | ,307^**^ | ,518^**^ | 0,042 | -0,038 | ,452^**^ | 0,015 | ,185^**^ | ,334^**^ | ,339^**^ | ,430^**^ | ,298^**^ | ,310^**^ | ,268^**^ | ,389^**^ | 0,113 | ,268^**^ | 1,000 |  |  |  |  |
| Threonine | ,402^**^ | ,214^**^ | ,397^**^ | 0,035 | ,327^**^ | ,132^*^ | 0,104 | ,182^**^ | ,396^**^ | ,288^**^ | ,126^*^ | 0,084 | ,371^**^ | ,415^**^ | ,225^**^ | 0,086 | ,358^**^ | ,539^**^ | 0,017 | 1,000 |  |  |  |
| Tryptophan | ,260^**^ | 0,048 | ,295^**^ | -0,043 | ,127^*^ | ,163^**^ | -0,053 | 0,015 | ,173^**^ | ,269^**^ | ,198^**^ | ,245^**^ | ,356^**^ | ,436^**^ | ,271^**^ | ,392^**^ | ,180^**^ | ,153^*^ | 0,008 | ,242^**^ | 1,000 |  |  |
| Tyrosine | ,484^**^ | ,147^*^ | ,406^**^ | ,160^**^ | ,389^**^ | 0,099 | 0,107 | ,233^**^ | ,309^**^ | ,339^**^ | ,354^**^ | ,382^**^ | ,441^**^ | ,532^**^ | ,449^**^ | ,469^**^ | ,434^**^ | ,332^**^ | ,153^*^ | ,260^**^ | ,365^**^ | 1,000 |  |
| Valine | ,138^*^ | 0,082 | ,209^**^ | ,247^**^ | ,238^**^ | 0,038 | ,159^**^ | -0,037 | -0,032 | ,390^**^ | ,789^**^ | ,840^**^ | ,492^**^ | ,273^**^ | ,253^**^ | ,576^**^ | ,260^**^ | ,155^*^ | ,211^**^ | ,196^**^ | ,330^**^ | ,395^**^ | 1,000 |

** - Correlation is significant at 0.01 level, * - Correlation is significant at 0.05 level
